# A Canvas of Spatially Arranged DNA Strands that Can Produce 24-bit Color Depth

**DOI:** 10.1021/jacs.3c06500

**Published:** 2023-10-03

**Authors:** Tadija Kekić, Jory Lietard

**Affiliations:** Institute of Inorganic Chemistry, University of Vienna, Josef-Holaubek-Platz 2, 1090 Vienna, Austria

## Abstract

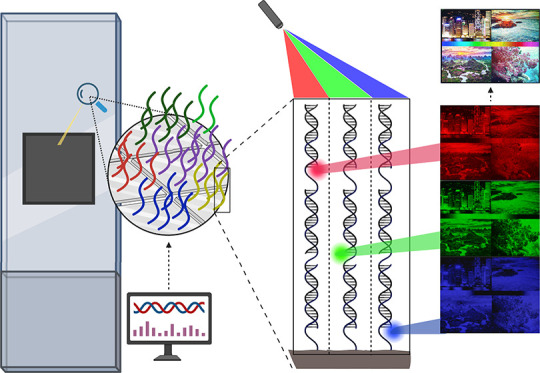

Nucleic acid microarray photolithography combines density, throughput, and positional control in DNA synthesis. These surface-bound sequence libraries are conventionally used in large-scale hybridization assays against fluorescently labeled, perfect-match DNA strands. Here, we introduce another layer of control for *in situ* microarray synthesis—hybridization affinity—to precisely modulate fluorescence intensity upon duplex formation. Using a combination of Cy3-, Cy5-, and fluorescein-labeled targets and an ensemble of truncated DNA probes, we organize 256 shades of red, green, and blue intensities that can be superimposed and merged. In so doing, hybridization alone is able to produce a large palette of 16 million colors or 24-bit color depth. Digital images can be reproduced with high fidelity at the micrometer scale by using a simple process that assigns sequence to any RGB value. Largely automated, this approach can be seen as miniaturized DNA-based painting.

Modern color display is structured around the emission of light at the three primary red, green, and blue channels, joined with the ability to modulate its intensity. The modulation of light intensity, and therefore the construction of color shades, is a function of electric current in LEDs and OLEDs, a derivative of metasurface structure,^1−3^ or, in the most historical sense, a skillful mélange of dyes and pigments. Fluorescent dyes cover the entire human visible spectrum of light, and employing fluorescence is the method of choice for an extremely vast panel of chemical and biochemical assays, particularly in the context of monitoring the self-assembling properties of nucleic acids.^4,5^ With surface-bound DNA, duplex formation can be detected by using fluorescence as a readout, an essential aspect of the experimental framework with DNA microarrays. DNA microarrays are an ensemble of nucleic acid sequences attached to a solid surface.^6^ Whether spotted or synthesized *in situ*,^7,8^ the essence of a microarray is to precisely assign position to a unique DNA. DNA hybridization to complementary strands can inform on gene expression levels in a cell sample, and most microarray-based applications revolve around detecting the presence or absence of a fluorescence signal.^9−11^ Here, fluorescence intensity is a function of thermal stability of the duplex,^12^ but the correlation of these two parameters has not been fully explored yet. Still, hybridization alone on patterned nucleic acid surfaces can create simple visual motifs^13−18^ using multiple dyes to produce color and/or mismatches and truncations to alter intensity. Large-scale probing of the hybridization landscape via the introduction of mismatches and deletions should result in a much richer palette of fluorescence intensities from which a complex color scale can be assembled. The fluorescence intensity of conjugated dyes is known to be affected by sequence too,^19−21^ and this has previously been exploited to create a monochrome scale of 256 colors.^22^ However, because the process employs a terminal dye labeling step during DNA synthesis, the approach is not easily amenable to multiplexing. In this paper, we show that fluorescence signal can be fine-tuned by lowering the melting temperature of a DNA duplex. This strategy is extensible over a large range of light intensity that can be dissected into 256 shades of color, in all red, green, and blue channels using appropriate Cy3, Cy5, and fluorescein dyes on complementary DNA strands. All channels are simultaneously accessible on a long, surface-bound DNA scaffold that can display any RGB value in 24-bit color depth, or >16 million colors, beyond the 8-bit maximum that was previously achievable on such platforms.^14,18^ Using photolithography, a canvas of high-density DNA microarrays containing up to 786 000 addressable features can be fabricated^23,24^ and used to reproduce graphical input of any common digital format with high fidelity.

In this context, the feature of a microarray is the basic pixel unit of the DNA facsimile, a 14 × 14 μm^2^ area assigned to a unique sequence, with a 1 μm gap between pixels. Each feature is populated with an ∼100-nt-long single-stranded template that can hybridize to three short probes labeled with 5′-Cy3, Cy5, and fluorescein. We chose two 25mers (R and B) and one 30mer sequence (G) as color mediators because of their very high binding signals in the presence of a full match. In order to design variations in affinity, the *T*_m_ of each hybridizable section of the RGB scaffold is tweaked by introducing deletions. In so doing, the fluorescence signal acts as a slider that can be controlled and adjusted between two extreme values corresponding to no match and full match (Figure 1). We limit the number of deletions to 4 per section, as we found that 4 strategically placed deletions are sufficient to completely abolish duplex formation in both 25- and 30mers. The total number of possible deletions is given in eq 1:
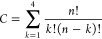
1where *C* is the number of combinations, *n* the probe length, and *k* the number of deletions. This yields ∼15 000 and ∼32 000 combinations of mismatched 25- and 30mers, respectively, all readily synthesized in parallel on microarrays with >10 technical replicates. Hybridization to either the R, G, or B library reveals a large range of fluorescence signals for each dye, contained between that of the full complement and that of a heavily impaired sequence (Figure 2A). As expected, 4 deletions scattered along the scaffold sequences yield very low fluorescence signals, close to the background (“black”). For all three sequences, deletions located at the extremities are on average much less detrimental to hybridization signal than centrally located deletions (Figure S1, Supporting Information). However, positions that are most sensitive to the introduction of deletions differ depending on the probe. This observation puts the emphasis on the nontrivial aspect of the relationship between mismatch and duplex affinity and the need for calibration. From the distribution of binding signals, we then aimed at selecting and constructing a range of linearly increasing fluorescence that can be further cut into 256 steps of equal size, or bins (Figure 2B). To ensure maximum contrast for each color channel, the widest range of fluorescence is necessary. Using a custom-built script, tail sequences of highest and lowest intensities were eliminated one by one, until each equidistant bin was populated with at least five sequences. This process was repeated for all three probes for a final number of bins of 768, or 3 × 256. With this approach, we assembled a calibration curve for all three color channels that uses >97% of the fluorescence range of the combinatorial libraries. Overall, this allocation process yielded a pool of >3800 unique DNA sequences that can encode any red, green, or blue value in the 0–255 range, or 16 million colors.

**Figure 1:**
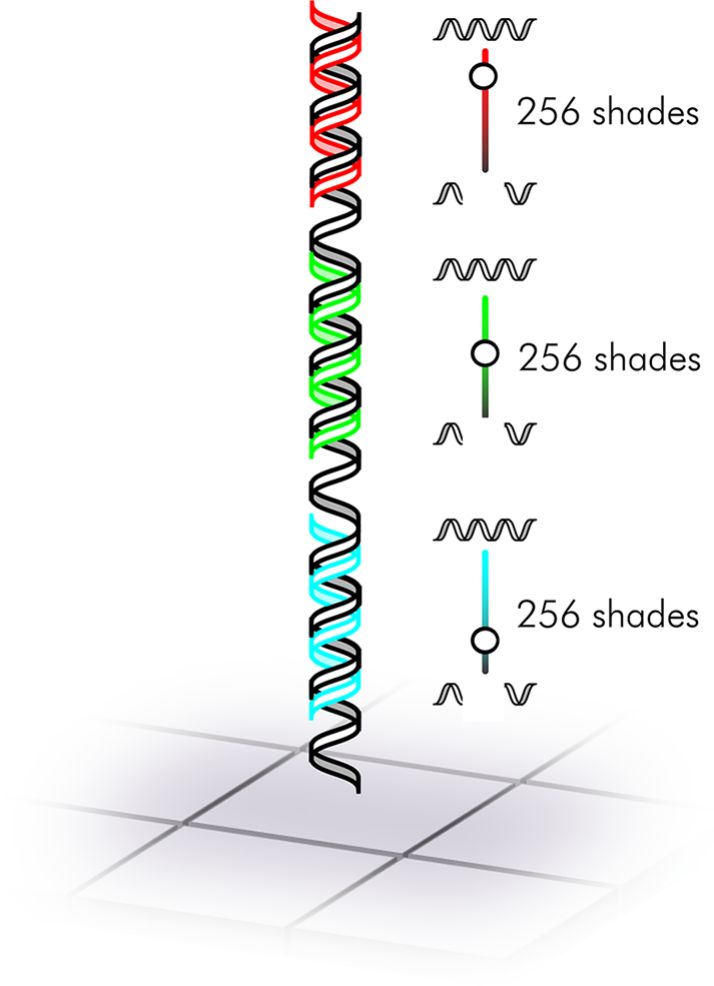
Schematic overview of the RGB scaffold used to perform simultaneous hybridization to red-, green-, and blue-labeled complementary probes. The hybridization affinity can be modulated by introducing deletions into the scaffold, lowering the fluorescence signal in 256 gradual steps for each R, G, and B channel.

**Figure 2:**
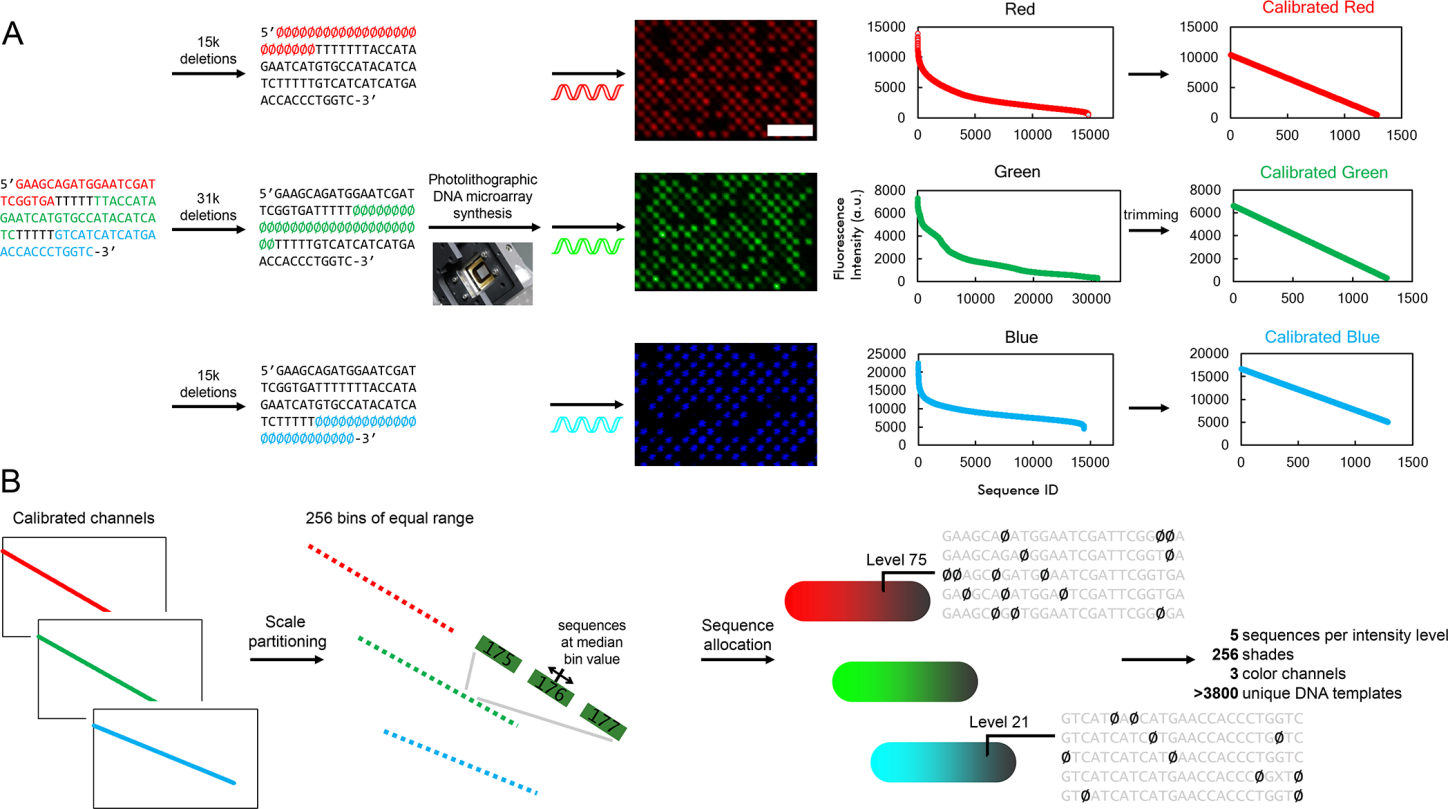
(A) Three libraries of DNA templates containing various amounts of deletions (15 000 or 31 000 possible shortmers at each hybridization stage, deletions noted as Ø) are synthesized as high-density DNA microarrays using maskless photolithography and hybridized to a Cy3-, Cy5-, or fluorescein-labeled probe. The distribution of fluorescence intensities is then trimmed down to linearity to generate calibration curves for all channels. Excerpts of scans represent <1% of the total area. Scale bar is ∼100 μm. (B) Process of sequence allocation to color intensity values. The fluorescence range in each RGB channel is divided into 256 bins spanning an equal intensity range. Within each bin, sequences close to median intensity value are chosen as representative of that particular color intensity. In practice, each level is populated with ∼5 unique DNA sequences containing a set amount of deletions at precise locations, for all 256 shades and for all three color channels, resulting in a library of >3800 DNA templates that can represent any color in a 24-bit RGB.

With this complete palette of DNA colors, we attempted to paint DNA microarrays using photolithography and maskless array synthesis (MAS). Through MAS, a pixel pattern can be imaged onto a glass surface, resulting in micrometer-size reproductions composed of square-like features. Imaging is carried out through reflection of light off a digital micromirror device (DMD). In microarray photolithography, light is 365 nm UV and serves to photodeprotect the 5′ end of the growing oligonucleotide chain. Only mirrors turned in an “ON” position will expose the corresponding features to UV. A pattern of “ON” and “OFF” mirrors therefore controls the spatial arrangement of sequences across the surface of the array but not the color composition itself.

To paint DNA on a microarray canvas, a full-color digital input must first be broken down into its three 8-bit RGB components (Figure 3). Each monochromatic layer now displays pixel values between 0 and 255. In a custom-made script, each color channel was graphed as a 1024 × 768 matrix of integers values ranging from 0 to 255, a size that corresponds to the resolution of the DMD. Elements of this matrix were semirandomly populated by one of five sequences allocated to the bins of the same value for that channel. This resulted in three sequence matrices, one complementary to each probe. The matrices were then linearized and merged to form 786 432 DNA sequences, where each sequence represents one pixel of the digital input. We adjusted the length of the polydT separators between the probe docking sections on the DNA template to compensate for deleted nucleotides. In so doing, all DNA sequences are 100 nt long, regardless of the number of deletions. Then, a second script transforms sequence and position into a series of digital masks and instructions for the automated synthesis of the oligonucleotide library. A checkered pattern of “ON” mirrors was chosen to increase synthesis quality and reduce cross-illumination of neighboring features,^25^ “OFF” mirrors being solely used to passivate the corresponding surface area with a capping agent (an acid-sensitive DMTr protecting group).

**Figure 3:**
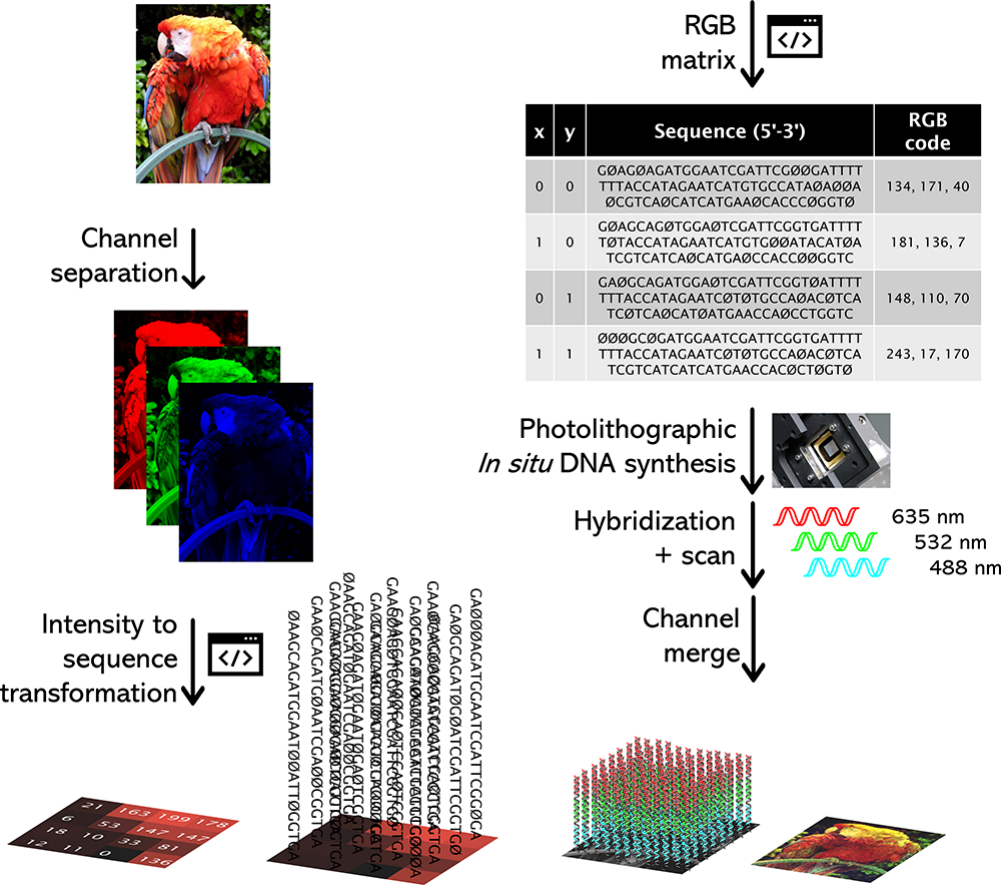
Reproduction process of a 24-bit color digital input into a DNA microarray of equal color depth. A script decomposes the input picture and assigns a DNA sequence for each intensity value in each red, green, and blue sublayer, conserving every pixel coordinate. A second script then merges the sequences together and creates the instructions for the synthesis of the corresponding oligonucleotides using phosphoramidite chemistry and MAS. Subsequent hybridization to three fluorescent probes, scanning at 635, 532, and 488 nm, and merging RGB channels then reveals a colored surface made of hybridized DNA templates. The parrot image has been released into the public domain by its creator, Psychonaut.

We took four 24-bit, public-domain digital images, with complex patterns and a large spectrum of color (Figure S2, Supporting Information) and injected them into our DNA photocopying process. The results are shown in Figure 4 and reveal fairly high color fidelity between the original and the DNA copy. The color palette in the middle section of the collage gives a good approximation of the reproduction accuracy. All colors and color transitions are present, with a few inaccuracies, most notably the imperfect orange section and the larger turquoise section. The low orange content is responsible for the incorrect depiction of the sunset at sea, which appears too yellow. Otherwise, visual details like waves, riverbed, and coral patterns are completely discernible. Likewise, brightness and contrast are preserved, with the waterfront being properly illuminated without saturation. Total brightness of the DNA painting appears higher than in the input, which can be ascribed to background fluorescence coming from nonspecific probe binding to the surface of the array. Upon closer inspection of the merged RGB channels (Figure S3, Supporting Information), the overall grainy aspect of the output image can be tracked down to the unexpected/exaggerated presence of red color in features that seem to dot the surface. Indeed, of all three color channels, the red color rendering is the least accurate (Figures S4, S5, and S6, Supporting Information), which would explain the incomplete orange portion of the color spectrum and the presence of yellow in the green section (G+ distorted R value). This phenomenon may be the result of positioning of the red probe on the DNA scaffold, the furthest away from the surface, which affects both DNA synthesis quality and fluorescence intensity due to the significant distance between dye and array surface. Changing the scaffolding order or, alternatively, selecting a more photostable red fluorescent dye^26^ should improve performance for this channel.

**Figure 4:**
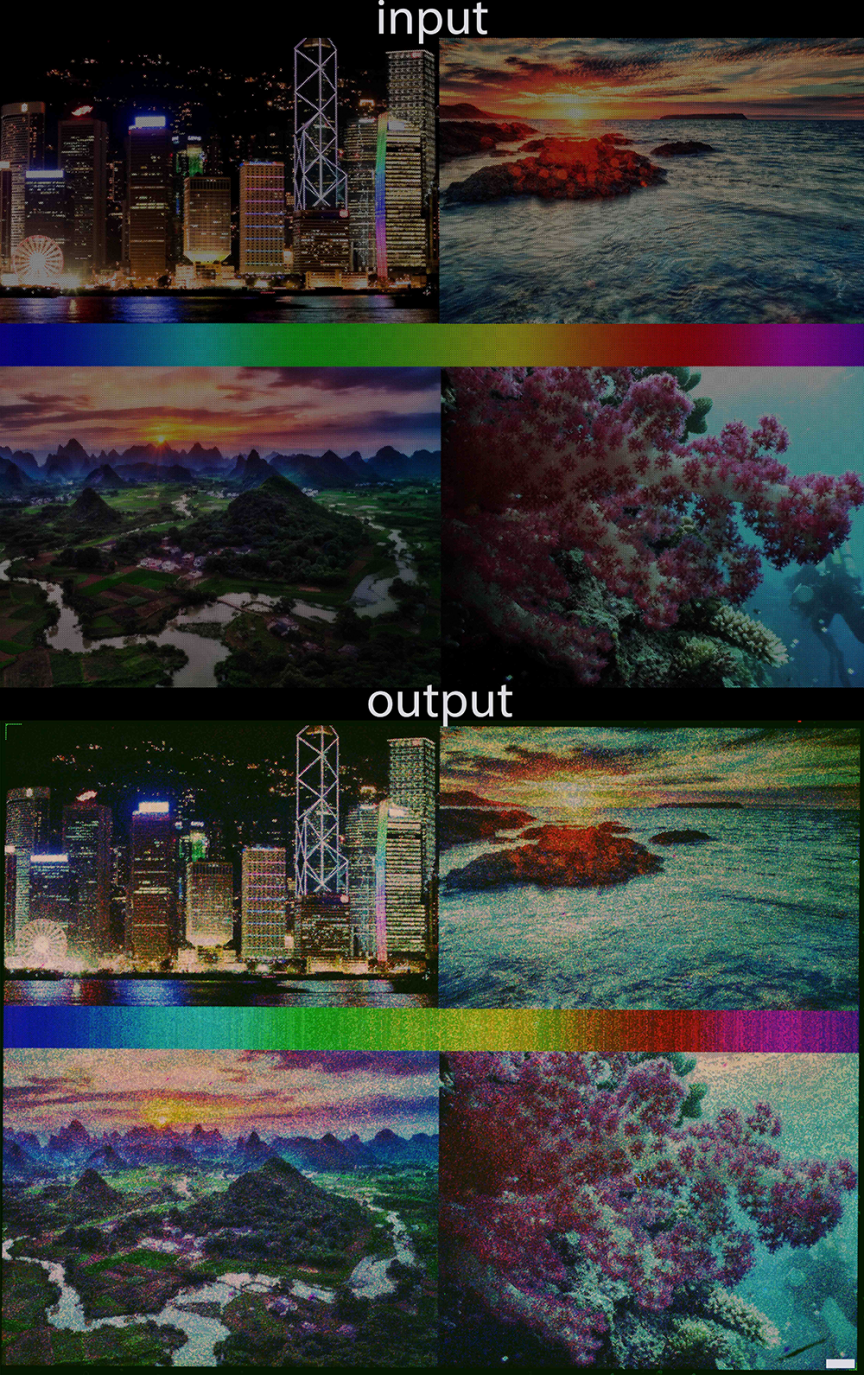
Reproduction in nucleic acid format of a 1024 × 768 collage of digital images in 24-bit color (top) and autocomposition of RGB channels from three microarray scans at 635, 532, and 488 nm (bottom). The microarray (1 × 1.4 cm in size) was scanned at 2.5 μm resolution. Scale bar is ∼500 μm. The following digital images: “Beautiful Hong Kong” by cblee, “Beautiful Guilin at Sunset” by Trey Ratcliff, “All the Colors” by stewartbaird, and “NOAA Ocean Explorer: Pacific Deep Reefs 2011 Exploration: Mission Summary” by NOAA Ocean Exploration & Research are licensed under CC BY-NC-SA 2.0. Source is available at openverse.org.

In summary, we have shown that we can control hybridization affinity in minute amounts, with multiple probes bound to a single DNA template. Fine-tuning hybridization was translated into a color scale, with probe multiplexing allowing for the creations of 16 million colors and the faithful reproduction of 24-bit digital pictures in a DNA microarray format. Beyond micrometer-sized biopolymer painting, this level of control in duplex affinity could be useful in biosensors and diagnostics and wherever the self-assembling properties of nucleic acids require delicate adjustment. The ability to generate precise color signals could also be the key to multiplexing surface-based assays, for instance, computational work with complex DNA chips.

## Supporting Information Available

List of sequences associated with each color value (XLSX)
Experimental details and supplementary figures (PDF)
Input RGB image as a high-resolution file (PDF)
Output RGB image as a high-resolution file is available at https://zenodo.org/record/8395197.

## Abbreviations

DMD: digital micromirror device
MAS: maskless array synthesis
