# Supplementary material for: A Canvas of Spatially Arranged DNA Strands that Can Produce 24-bit Color Depth
Source: J Am Chem Soc. 2023 Oct 3;145(41):22293–7. doi: 10.1021/jacs.3c06500 (PMC10591465; doi:10.1021/jacs.3c06500)
Supplement: Supplementary file 2 — ja3c06500_si_002.pdf [file ja3c06500_si_002.pdf]

Supporting Information

A Canvas of Spatially Arranged DNA Strands that can Produce 24-bit Color Depth

Tadija Kekić, Jory Lietard*

Institute of Inorganic Chemistry, Faculty of Chemistry, University of Vienna, Josef-Holaubek-Platz 2 (UZA II), 1090 Vienna, Austria

* Correspondence: jory.lietard@univie.ac.at

Table of Contents

1. Probe sequences and complementary DNA sequences on microarray........................2
2. Principle of maskless microarray photolithography..2
3. DNA microarray synthesis and conditions 3
4. Hybridization, scanning and data extraction 4
5. Channel calibration and sequence assignment 5
6. Microarray design for digital input reproduction 6
7. Channel merge..7
8. Scripts - Image2Sequence converter...7
9. Supplementary figures 13

1. Probe sequences and complementary DNA sequences on microarray

Supplementary Table 1. Sequences of oligonucleotides, complementary to red, green and blue domain, terminally labelled with respective fluorophores, used as probes in RGB-Calibration and RGB-Imaging experiments.

#	Probe name	Color Domain	Sequence (5' to 3')	Mod	Chem	Length
**1**	Click-Cy5_DNA	Red	UCA CCG AAU CGA UUC CAU CUG CUU C	5'-Cy5	RNA	25
**2**	GLD1-Cy3_DNA	Green	GAT GAT GTA TGG CAC ATG ATT CTA TGG TAA	5'-Cy3	DNA	30
**3**	QC25-FAM_DNA	Blue	GAC CAG GGT GGT TCA TGA TGA TGA C	5'-FAM	DNA	25

Supplementary Table 2. References for sequence variations in color domains. Domains were separated by poly-dT spacers and synthesised in series Blue-Green-Red (3'⟶5') on the surface of the microarray.

#	Complementary to Probe	Color Domain	Base Array Sequence (5' to 3')	Variations	Chem	Mutation Type
**1**	Click-Cy5_DNA	Red	G AAG CAG ATG GAA TCG ATT CGG TGA	15 275	DNA	Truncation
**2**	GLD1-Cy3_DNA	Green	TTA CCA TAG AAT CAT GTG CCA TAC ATC ATC	31 930	DNA	Truncation
**3**	QC25-FAM_DNA	Blue	G TCA TCA TCA TGA ACC ACC CTG GTC	15 275	DNA	Truncation

2. Principle of maskless microarray photolithography

Modern nucleic acid synthesis conventionally uses phosphoramidite chemistry and a cycle-based approach to oligonucleotide elongation. In the cyclic process, the deprotection of the 5' hydroxyl group of the growing oligonucleotide strands is followed by the coupling of a pre-activated nucleoside phosphoramidite, taking place in the reaction chamber. Each growing oligonucleotide with an available terminal hydroxyl is coupled with a single activated phosphoramidite. The product of reaction, a phosphite triester, is then oxidized and the next cycle is initiated.

In the most conventional method, oligonucleotide synthesis is carried out on solid support, usually in the form control pore glass beads (CPGs) loaded onto a reaction chamber. The reaction chamber is connected to the chemical delivery system and each cycle begins with a

5'-OH deblocking by flushing an acidic solution. As such this method allows for the synthesis of only one sequence per chamber. By exchanging the standard acid sensitive protection group for a light sensitive NPPOC (nitrophenylpropyloxycarbonyl) group the reaction cycle can be initiated using 365 nm UV light exposure in presence of a weak organic base. This photodeprotection step can be spatially controlled using photolithography.

The instrumentation that regulates both the delivery of chemicals and controls patterns of light exposure is called Maskless Array Synthesizer (MAS). In MAS setup, the spatially controlled light exposure is carried out by a digital micromirror device (DMD). The total number of unique sequences or microarray features is equal to the number of individually controlled mirrors in the DMD. In our setup, we use an 0.7" XGA DMD (Texas Instruments) with 786 432 micromirrors (1024 x 768 resolution). The light from 365 nm high-power UV-LED (Nichia NVSU333A) is first spatially homogenized in a light pipe before reaching the DMD device. The "ON" mirrors of the DMD reflects incoming light onto an Offner imaging system. The light is reflected from the Offner relay onto the surface of the microarray reaction chamber. "OFF" mirrors reflect UV light away from the surface of the array. The microarray reaction chamber is comprised of two microscope glass slides (Schott Glass D) pre-functionalised with *N*-(3-triethoxysilylpropyl)-4-hydroxybutyramide (Gelest SIT8189.5). Slides are separated by a 50-µm thick Teflon gasket forming a microfluidic chamber. One microarray slide is pre-drilled on a CNC router (Stepcraft) at appropriate positions matching the inlet and outlet tubing positions of the quartz block of the reaction chamber on which the microscope slides are deposited. Inlet and outlet microfluidic lines are then connected to an automated nucleic acid synthesizer (Expedite 8909, Perseptive Biosystems).

3. DNA microarray synthesis and conditions

Our setup relies on use of the anhydrous acetonitrile (ACN) (Sigma-Aldrich) as a wash solvent between chemical reactions. The A / C / G / T DNA phosphoramidites (Orgentis) are photoprotected with a 5' BzNPPOC (benzyl-NPPOC) group and were dissolved in ACN to a final concentration of 0.03 M. Phosphoramidites were activated with 0.25 M 4,5-dicyanoimidazole in ACN (Sigma-Aldrich). The coupling time for all phosphoramidites was 15 s. The light-facilitated deprotection reaction was conducted in 1 % (w/v) imidazole (Sigma-Aldrich 56750) in anhydrous DMSO (Sigma-Aldrich). The time of light exposure reaction was proportional to the current average exposure strength of the light source (~ 80 mW/cm^2) and was approximately 37.5 s. This exposure time is adjusted so that the total radiant exposure of 3 J/cm^2 was reached, equating to ~95% photodeprotection efficiency. UV power was

measured on a UV detector with 365 nm probe (SÜSS MicroTec, model 1000). The oxidation reaction was done using a solution of tetrahydrofuran / water / pyridine / iodine in 90.54 / 9.05 / 0.41 / 0.43 (v / v / v / w) (Sigma-Aldrich L5860021). The time of oxidation reaction was 30 s in each cycle and 200 s at the end of the synthesis.

Following the synthesis, the deprotection was performed by incubating the slides in 1:1 (v/v) ethylenediamine/ethanol for two hours at room temperature. Slides were thoroughly washed in ddH_2O, dried by centrifugation, and stored in dry conditions until further use.

4. Hybridization, scanning and data extraction

Following the complete deprotection, a self-adhesive hybridisation chamber (Grace Biolabs SA200) were placed over the microarray. The chamber was filled with 300 µl of hybridization buffer. Buffer consists of 150 µl 2x MES buffer (100 mM MES, 1 M Na^+, 20 mM EDTA, 0.01 % Tween20), 110 µl of nuclease free water (Carl Roth), 13.3 µl of acetylated BSA (10 mg/ml, Promega) and 26.7 µl of the 100 nM probe oligonucleotide (Supplementary Table 1.). The incubation is done for 2 h at 42 °C with rotation in a hybridisation oven (Boekel Scientific). Following hybridisation, the microarray slides were washed in three washing buffers with strong agitation. First, for 2 min in non-stringent wash buffer (0.9 M NaCl, 0.06 M sodium phosphate, 6 mM EDTA, 0.01 % Tween20); second, for 1 min in stringent wash buffer (100 mM MES, 0.1 M NaCl, 0.01 % Tween20); third, for 5 s in 0.1 x SSC. Slides were dried by centrifugation and scanned on a microarray scanner (GenePix 4400A). Slides were scanned using the red (635 nm), green (532 nm) or blue laser (488 nm) at 2.5 µm scanning resolution.

To regenerate the microarrays, probes were separated from the array by incubating in ddH_2O under 75 °C for 10 min. The cross-hybridization effect was analysed by rehybridization of the arrays with other two, non-complementary probes, separately.

Data extraction from microarray scans was done using NimbleScan 2.1.68 software (Nimblegen). The image file, containing the scanned microarray, and the matrix file, containing the intended sequence identifiers for all pixels used in the construction of digital masks for the MAS were overlayed. The matrix file was manually aligned to the image with the help of corner fiducials containing positive control sequences. For each feature, NimbleScan calculates median and standard error and allocates the sequence identifier. Initial data processing was finalized by a custom python script (Flash_v3) suited for average, median and standard error calculation from features with the same sequence identifier.

5. Channel calibration and sequence assignment

The Stacked Calibration microarray allowed for the calibration of complementary sequences where all three color domains were synthesized on top of one another, in R->G->B orientation. In the linear model of this design, the blue domain was closest to the surface and red domain was most remote. Each color domain was preceded by a dT_5 spacer extended by a number of additional T equal to the number of truncations in the sequence of the following color domain. In the linear model, this extension of the dT_5 spacer maintains the relative distance both between fluorophores themselves and between fluorophores and the surface of the array. The Stacked Calibration design closely resembles the real RGB painting design and it allows for recognition of sequences prone to cross-hybridization and other types of less predictable inter and intra-molecular interactions, specific to some variations of the 100nt long oligonucleotides. The elimination of these sequences from further processing is necessary to establish stable and reproducible fluorescent signal in RGB painting.

The Stacked Calibration array facilitates the sequence calibration for all three probes. This is achieved by making two domains of the RGB oligonucleotides fully complementary to their respective probes, while the third domain is variable. The sheer number of sequence variations in combination with their 100nt length makes the synthesis of stacked calibration array very time consuming. This is further emphasized by the fact that usual synthesis optimization protocols, based on the reduction of number of phosphoramidite cycles in the synthesis, fail to work with combinatorial introduction of deletions. By refining the synthesis optimization procedure, using multiple sequence alignment, each deletion was treated as a dummy cycle so the deletions would not cause loss of the synchronisation of otherwise almost identical synthesis. In doing so we saved 75% of synthesis time and reagent consumption. This allowed for synthesis of 62 480 unique, 100nt long DNA oligonucleotides in only 108 cycles and 3.5 hours. Each sequence had 12 technical replicates per microarray slide, and 24 replicates in total.

This works particularly well if we choose to focus on deletions only. Such an approach would not be possible if we were to probe the mismatch space instead. The total number of combinations of sequences that can contain up to four mismatches is, as mentioned for the total number of sequences containing four deletions, given by the following equation:

$$C = \sum_{k=1}^{4} \frac{n!}{k!(n-k)!}$$

Or ~15000 combinations in a 25-nt long sequence. However, these are the number of possible combinations with up to four mismatches scattered along the sequence, not the actual number of sequences. At each position accepting a mismatch there are three possibilities, for instance C, G or T if the corresponding position initially contains an A. Therefore, the total number of sequences with up to four mismatches become 15000 × 4^4, which is ~4 million. A curation of these sequences to keep within our microarray capabilities would be possible, but it would still translate into a very long synthesis time. Indeed, with each position along the 100-nt template now accepting any of the four nucleobases, the photolithography protocol would be forced to follow a A⟶C⟶G⟶T amidite coupling cycle, pushing the total number of synthesis cycles at ~400. This would correspond to ~14 h of synthesis time. Some curation could be carried out at this level too and sequences that either too T-rich or running against the A⟶C⟶G⟶T cycle could be omitted, for a reduction of the total number of coupling cycles, but this is likely to only reduce it by a maximum number of ~50. For convenience, practicality and for the sake of completeness in our assays, we chose to focus on deletions only.

After synthesis and hybridization to the deletion library for calibration purposes, sequence assignment was conducted in two main steps. In the first step, for each probe, sequences with variations in complementary color domain were sorted by their signal strength. In three rounds of elimination, sequences that had standard error higher than the median of errors for sequences within the domain were eliminated. Sequences were normalized and separated into 256 subsets or bins, equidistant by their median signal. In the recursive process, tail sequences, of highest and lowest intensities, were eliminated one by one until almost all equidistant bins were populated with at least 5 sequences. Due to the standard distribution of sequence dependent intensities, the central bins were populated with much more candidate sequences than the highest and lowest bins. Since this trimming process reduced the signal intensity range for each probe, when deemed reasonable we allowed for less than 5 sequences in few highest and lowest bins of each color domain. In the second step, five best sequence candidates, chosen by their proximity to the median value of each bin, were kept and the rest of the sequences were discarded. This process was repeated for all three probes with final number of bins being 768, or 3 x 256.

6. Microarray design for digital input reproduction

The chosen digital image in 24-bit RGB format, with a resolution of 1024 x 768 was deconstructed in three 8-bit color channels and exported in .bmp format using Gimp 2.10.24 imaging software. In custom made python script (Image2Sequence converter), each channel

was represented by the 1024 x 768 matrix of integers values ranging from 0 to 256, representing shades of red, green, or blue color, depending on the channel. The integer matrices were used as a reference for construction of a sequence matrices of the same size. This is achieved by exchanging each element of the integer matrix with one of five sequences, semi-randomly chosen from previously allocated bins for that channel. This resulted in three sequence matrices, each complementary to one probe, where number of unique sequences per matrix would be up to 1280 or 5 x 256. Following construction, matrices were linearized and conjoined to form 786 432 sequences, each 100nt in length, where each sequence represents one pixel of the chosen digital image. To increase photolithographic quality during the synthesis, a checkered pattern of features was overlaid over the sequences, meaning that every second sequence of the array was exchanged for a passivation cap, meaning no synthesis. Finally, corner pixels were exchanged for fully complementary probe sequences to act as fiducials elements, as required for data extraction. Now completed and linearized, the sequence matrix was optimized using multiple sequence alignment and used as the input file in custom-made MATLAB script for digital mask generation. The MATLAB script and all steps required for creation of a synthesis ready instruction files were extensively described earlier.

7. Channel merge

Synthesis, deprotection, hybridisation and scanning of microarrays containing pictorial information in 24bit RGB format followed all steps previously described in the SI. High-resolution scans of microarray under three different laser-filter combinations were opened in Gimp 2.10.24 imaging software. They were overlaid and aligned based on corner fiducials containing fully complementary control sequences. Contrast and brightness of each scan was auto adjusted as to have range between 0 and 1 with hue preservation. Following this, three scans, each designated to its respective channel were automatically merged into a single image in 24bit RGB format.

8. Scripts - Image2Sequence converter

```
from PIL import Image
import numpy as np
import random
#Required Files:
#SeqRange_Red.txt
#SeqRange_Green.txt
#SeqRange_Blue.txt
#SelectedSeqGradRange.txt
```

```
#SelectedSeqGradIndex.txt
#SelectedSeqGradCount.txt
#Red_Adobe.bmp
#Green_Adobe.bmp
#Blue_Adobe.bmp
#SeqDepF_Fedu cials.bmp
#SeqDepF_Layout.bmp
# THIS TAKES SINGLE LINED TXT FILE OF BLUE SEQUENCES WITH NAMES (RANKED FROM LOWEST TO HIGHEST) AND MAKES AN ARRAY
OUT OF IT
print("Program Initialised")
print("Loading Images")
RealSeqRange = np.loadtxt("SeqRange_Blue.txt", dtype=str)
RealSeqRange = RealSeqRange.reshape((len(RealSeqRange), 1), order='F')
RealSeqIndex = np.loadtxt("SeqIndex.txt", dtype=str)
RealSeqIndex = RealSeqIndex.reshape((len(RealSeqIndex), 1), order='F')
RealSeqCount = np.loadtxt("SeqCount.txt", dtype=str)
RealSeqCount = RealSeqCount.reshape((len(RealSeqCount), 1), order='F')
SequenceListFinal = [None] * (256*5)  # from 256
random.seed(1)
countA = 0
countB = 0
CountC = 0
for i in range(256):
    countB = countB + (int(str(RealSeqCount[i])[2:-2])-1) # -1 is new to fix !
    y1 = random.choice(range(countA, countB))
    y2 = random.choice(range(countA, countB))
    y3 = random.choice(range(countA, countB))
    y4 = random.choice(range(countA, countB))
    y5 = random.choice(range(countA, countB))
    countA = countB + 1
    countB = countB + 1  # new to fix !
    SequenceListFinal[CountC] = str(RealSeqRange[y1])[2:-2]
    CountC = CountC + 1
    SequenceListFinal[CountC] = str(RealSeqRange[y2])[2:-2]
    CountC = CountC + 1
    SequenceListFinal[CountC] = str(RealSeqRange[y3])[2:-2]
    CountC = CountC + 1
    SequenceListFinal[CountC] = str(RealSeqRange[y4])[2:-2]
    CountC = CountC + 1
    SequenceListFinal[CountC] = str(RealSeqRange[y5])[2:-2]
    CountC = CountC + 1
with open('BinnedSeq8bit.txt', 'w') as file_handle:
    for list_item in SequenceListFinal:
        file_handle.write('%s\n' % list_item)
# THIS OPENS INPUT TIFF FILE AS ARRAY
Base = Image.open('Blue_Adobe.bmp')
Base = np.array(Base)
# THIS MODIFIES INPUT ARRAY IN A SINGLE LINE FILE GOING FROM 0-768 (for 1024 times)
grid_Base = (Base.reshape((786432, 1), order='F'))
# THIS SAVES THE GRID FILE AS TXT AND OPENS IT AS A LIST
np.savetxt("single_line_Base.txt", grid_Base, delimiter="", fmt='%5.0f')
Sequence_Base = np.array(grid_Base)
Sequence_Base = Sequence_Base.astype('str')
Sequence_BaseList = Sequence_Base.tolist()
# THIS TAKES SINGLE LINED TXT FILE OF SEQUENCES WITH NAMES (RANKED FROM LOWEST TO HIGHEST) AND MAKES AN ARRAY OUT OF
IT
RealSeq = np.loadtxt("BinnedSeq8bit.txt", dtype=str)
RealSeq = RealSeq.reshape((len(RealSeq), 1), order='F')
# SEED IS NOT NEEDED BUT STAYS FOR TESTING JUST IN CASE
random.seed(1)
# THIS LOOPS OVER ARRAY TO MATCH GRAYSCALE PIXEL VALUE-LOCATION TO ALLOCATED SEQUENCE (per SEQ MAP)
# AND EXPORT A FILE READY FOR INPUT IN MATLAB PROGRAM
SequenceListFinal = Sequence_BaseList
x = (RealSeq.tolist())
j = 0
for i in Sequence_BaseList:
```

```
    y = random.choice(range((int(str(i)[2:-2])*5), ((int(str(i)[2:-2])*5)+5)))
    SequenceListFinal[j] = str(x[y])[2:-2]#was 3:-2
    j = j + 1
# THIS ADDS FEDUCIALS FOR EACH SECTION OF FULL SEQUENCE
Feducials = Image.open("SeqDepF_Feducials.bmp")
Feducials = np.array(Feducials)
# THIS MODIFIES INPUT ARRAY IN A SINGLE LINE FILE GOING FROM 0-768 (for 1024 times)
grid_Feducials = (Feducials.reshape((786432,1), order = "F"))
# THIS SAVES THE GRID FILE AS TXT AND OPENS IT AS A LIST
np.savetxt("single_line_Feducials.txt", grid_Feducials, delimiter="", fmt='%5.0f')
Sequence_Feducials = np.array(grid_Feducials)
Sequence_Feducials = Sequence_Feducials.astype("str")
Sequence_FeducialsList = Sequence_Feducials.tolist()
RealSeqFeducials = np.loadtxt("SelectedFeducials.txt", dtype=str)
RealSeqFeducials = RealSeqFeducials.reshape((len(RealSeqFeducials), 1), order = 'F')
x_Feducials = (RealSeqFeducials.tolist())
for i in range(len(Sequence_FeducialsList)):
    if str(Sequence_FeducialsList[i])[2:-2] == str(255):
        SequenceListFinal[i] = str(x_Feducials[0])[2:-2]
# THIS ADDS PASSIVATION PATTERN TO THE ARRAY
Layout = Image.open("SeqDepF_Layout.bmp")
Layout = np.array(Layout)
grid_Layout = (Layout.reshape((786432,1), order = "F"))
np.savetxt("single_line_Layout.txt", grid_Layout, delimiter="", fmt='%5.0f')
Sequence_Layout = np.array(grid_Layout)
Sequence_Layout = Sequence_Layout.astype("str")
Sequence_LayoutList = Sequence_Layout.tolist()
RealSeqLayout = np.loadtxt("SelectedLayout.txt", dtype=str)
RealSeqLayout = RealSeqLayout.reshape((len(RealSeqLayout), 1), order = 'F')
x_Layout = (RealSeqLayout.tolist())
for i in range(len(Sequence_LayoutList)):
    if str(Sequence_LayoutList[i])[2:-2] == str(255):
        SequenceListFinal[i] = str(x_Layout[0])[2:-2]
# THIS CREATES INPUT FOR THE MATLAB
with open('SeqForMatlab8bit.txt', 'w') as file_handle:
    for list_item in SequenceListFinal:
        file_handle.write('%s\n' % list_item)
# THIS TAKES SINGLE LINED TXT FILE OF GREEN SEQUENCES WITH NAMES (RANKED FROM LOWEST TO HIGHEST) AND MAKES AN ARRAY
OUT OF IT
RealSeqRange = np.loadtxt("SeqRange_Green.txt", dtype=str)
RealSeqRange = RealSeqRange.reshape((len(RealSeqRange), 1), order='F')
RealSeqIndex = np.loadtxt("SeqIndex.txt", dtype=str)
RealSeqIndex = RealSeqIndex.reshape((len(RealSeqIndex), 1), order='F')
RealSeqCount = np.loadtxt("SeqCount.txt", dtype=str)
RealSeqCount = RealSeqCount.reshape((len(RealSeqCount), 1), order='F')
SequenceListFinal = [None] * (256*5)  # from 256
random.seed(1)
countA = 0
countB = 0
CountC = 0
for i in range(256):
    countB = countB + (int(str(RealSeqCount[i])[2:-2])-1) # -1 is new to fix !
    y1 = random.choice(range(countA, countB))
    y2 = random.choice(range(countA, countB))
    y3 = random.choice(range(countA, countB))
    y4 = random.choice(range(countA, countB))
    y5 = random.choice(range(countA, countB))
    countA = countB + 1
    countB = countB + 1  # new to fix !
    SequenceListFinal[CountC] = str(RealSeqRange[y1])[2:-2]
    CountC = CountC + 1
    SequenceListFinal[CountC] = str(RealSeqRange[y2])[2:-2]
    CountC = CountC + 1
    SequenceListFinal[CountC] = str(RealSeqRange[y3])[2:-2]
    CountC = CountC + 1
    SequenceListFinal[CountC] = str(RealSeqRange[y4])[2:-2]
```

```
    CountC = CountC + 1
    SequenceListFinal[CountC] = str(RealSeqRange[y5])[2:-2]
    CountC = CountC + 1
with open('BinnedSeq8bit.txt', 'w') as file_handle:
    for list_item in SequenceListFinal:
        file_handle.write('%s\n' % list_item)
# THIS OPENS INPUT TIFF FILE AS ARRAY
Base = Image.open('Green_Adobe.bmp')
Base = np.array(Base)
# THIS MODIFIES INPUT ARRAY IN A SINGLE LINE FILE GOING FROM 0-768 (for 1024 times)
grid_Base = (Base.reshape((786432, 1), order='F'))
# THIS SAVES THE GRID FILE AS TXT AND OPENS IT AS A LIST
np.savetxt("single_line_Base.txt", grid_Base, delimiter="", fmt='%5.0f')
Sequence_Base = np.array(grid_Base)
Sequence_Base = Sequence_Base.astype('str')
Sequence_BaseList = Sequence_Base.tolist()
# THIS TAKES SINGLE LINED TXT FILE OF SEQUENCES WITH NAMES (RANKED FROM LOWEST TO HIGHEST) AND MAKES AN ARRAY OUT OF
IT
RealSeq = np.loadtxt("BinnedSeq8bit.txt", dtype=str)
RealSeq = RealSeq.reshape((len(RealSeq), 1), order='F')
# SEED IS NOT NEEDED BUT STAYS FOR TESTING JUST IN CASE
random.seed(1)
# THIS LOOPS OVER ARRAY TO MATCH GRAYSCALE PIXEL VALUE-LOCATION TO ALLOCATED SEQUENCE (per SEQ MAP)
# AND EXPORT A FILE READY FOR INPUT IN MATLAB PROGRAM
SequenceListFinal = Sequence_BaseList
x = (RealSeq.tolist())
j = 0
for i in Sequence_BaseList:
    y = random.choice(range((int(str(i)[2:-2])*5), ((int(str(i)[2:-2])*5)+5)))
    SequenceListFinal[j] = str(x[y])[2:-2]#was 3:-2
    j = j + 1
# THIS ADDS FEDUCIALS FOR EACH SECTION OF FULL SEQUENCE
Fefucials = Image.open("SeqDepF_Feducials.bmp")
Feducials = np.array(Feducials)
# THIS MODIFIES INPUT ARRAY IN A SINGLE LINE FILE GOING FROM 0-768 (for 1024 times)
grid_Feducials = (Feducials.reshape((786432,1), order = "F"))
# THIS SAVES THE GRID FILE AS TXT AND OPENS IT AS A LIST
np.savetxt("single_line_Feducials.txt", grid_Feducials, delimiter="", fmt='%5.0f')
Sequence_Feducials = np.array(grid_Feducials)
Sequence_Feducials = Sequence_Feducials.astype("str")
Sequence_FeducialsList = Sequence_Feducials.tolist()
RealSeqFeducials = np.loadtxt("SelectedFeducials.txt", dtype=str)
RealSeqFeducials = RealSeqFeducials.reshape((len(RealSeqFeducials), 1), order = 'F')
x_Feducials = (RealSeqFeducials.tolist())
for i in range(len(Sequence_FeducialsList)):
    if str(Sequence_FeducialsList[i])[2:-2] == str(255):
        SequenceListFinal[i] = str(x_Feducials[0])[2:-2]
# THIS ADDS PASSIVATION PATTERN TO THE ARRAY
Layout = Image.open("SeqDepF_Layout.bmp")
Layout = np.array(Layout)
grid_Layout = (Layout.reshape((786432,1), order = "F"))
np.savetxt("single_line_Layout.txt", grid_Layout, delimiter="", fmt='%5.0f')
Sequence_Layout = np.array(grid_Layout)
Sequence_Layout = Sequence_Layout.astype("str")
Sequence_LayoutList = Sequence_Layout.tolist()
RealSeqLayout = np.loadtxt("SelectedLayout.txt", dtype=str)
RealSeqLayout = RealSeqLayout.reshape((len(RealSeqLayout), 1), order = 'F')
x_Layout = (RealSeqLayout.tolist())
for i in range(len(Sequence_LayoutList)):
    if str(Sequence_LayoutList[i])[2:-2] == str(255):
        SequenceListFinal[i] = str(x_Layout[0])[2:-2]
# THIS CREATES INPUT FOR THE MATLAB
with open('SeqForMatlab8bit.txt', 'w') as file_handle:
    for list_item in SequenceListFinal:
        file_handle.write('%s\n' % list_item)
```

```
# THIS TAKES SINGLE LINED TXT FILE OF GREEN SEQUENCES WITH NAMES (RANKED FROM LOWEST TO HIGHEST) AND MAKES AN ARRAY
OUT OF IT
RealSeqRange = np.loadtxt("SeqRange_Red.txt", dtype=str)
RealSeqRange = RealSeqRange.reshape((len(RealSeqRange), 1), order='F')
RealSeqIndex = np.loadtxt("SeqIndex.txt", dtype=str)
RealSeqIndex = RealSeqIndex.reshape((len(RealSeqIndex), 1), order='F')
RealSeqCount = np.loadtxt("SeqCount.txt", dtype=str)
RealSeqCount = RealSeqCount.reshape((len(RealSeqCount), 1), order='F')
SequenceListFinal = [None] * (256*5)  # from 256
random.seed(1)
countA = 0
countB = 0
CountC = 0
for i in range(256):
   countB = countB + (int(str(RealSeqCount[i])[2:-2])-1) # -1 is new to fix !
   y1 = random.choice(range(countA, countB))
   y2 = random.choice(range(countA, countB))
   y3 = random.choice(range(countA, countB))
   y4 = random.choice(range(countA, countB))
   y5 = random.choice(range(countA, countB))
   countA = countB + 1
   countB = countB + 1  # new to fix !
   SequenceListFinal[CountC] = str(RealSeqRange[y1])[2:-2]
   CountC = CountC + 1
   SequenceListFinal[CountC] = str(RealSeqRange[y2])[2:-2]
   CountC = CountC + 1
   SequenceListFinal[CountC] = str(RealSeqRange[y3])[2:-2]
   CountC = CountC + 1
   SequenceListFinal[CountC] = str(RealSeqRange[y4])[2:-2]
   CountC = CountC + 1
   SequenceListFinal[CountC] = str(RealSeqRange[y5])[2:-2]
   CountC = CountC + 1
with open('BinnedSeq8bit.txt', 'w') as file_handle:
   for list_item in SequenceListFinal:
      file_handle.write('%s\n' % list_item)
# THIS OPENS INPUT TIFF FILE AS ARRAY
Base = Image.open('Red_Adobe.bmp')
Base = np.array(Base)
# THIS MODIFIES INPUT ARRAY IN A SINGLE LINE FILE GOING FROM 0-768 (for 1024 times)
grid_Base = (Base.reshape((786432, 1), order='F'))
# THIS SAVES THE GRID FILE AS TXT AND OPENS IT AS A LIST
np.savetxt("single_line_Base.txt", grid_Base, delimiter="", fmt='%5.0f')
Sequence_Base = np.array(grid_Base)
Sequence_Base = Sequence_Base.astype('str')
Sequence_BaseList = Sequence_Base.tolist()
# THIS TAKES SINGLE LINED TXT FILE OF RED SEQUENCES WITH NAMES (RANKED FROM LOWEST TO HIGHEST) AND MAKES AN ARRAY OUT
OF IT
RealSeq = np.loadtxt("BinnedSeq8bit.txt", dtype=str)
RealSeq = RealSeq.reshape((len(RealSeq), 1), order='F')
# SEED IS NOT NEEDED BUT STAYS FOR TESTING JUST IN CASE
random.seed(1)
# THIS LOOPS OVER ARRAY TO MATCH GRAYSCALE PIXEL VALUE-LOCATION TO ALLOCATED SEQUENCE (per SEQ MAP)
# AND EXPORT A FILE READY FOR INPUT IN MATLAB PROGRAM
SequenceListFinal = Sequence_BaseList
x = (RealSeq.tolist())
j = 0
for i in Sequence_BaseList:
   y = random.choice(range((int(str(i)[2:-2])*5), ((int(str(i)[2:-2])*5)+5)))
   SequenceListFinal[j] = str(x[y])[2:-2]#was 3:-2
   j = j + 1
# THIS ADDS FEDUCIALS FOR EACH SECTION OF FULL SEQUENCE
Feducials = Image.open("SeqDepF_Feducials.bmp")
Feducials = np.array(Feducials)
# THIS MODIFIES INPUT ARRAY IN A SINGLE LINE FILE GOING FROM 0-768 (for 1024 times)
grid_Feducials = (Feducials.reshape((786432,1), order = "F"))
# THIS SAVES THE GRID FILE AS TXT AND OPENS IT AS A LIST
```

```
np.savetxt("single_line_Feducials.txt", grid_Feducials, delimiter="", fmt='%5.0f')
Sequence_Feducials = np.array(grid_Feducials)
Sequence_Feducials = Sequence_Feducials.astype("str")
Sequence_FeducialsList = Sequence_Feducials.tolist()
RealSeqFeducials = np.loadtxt("SelectedFeducials.txt", dtype=str)
RealSeqFeducials = RealSeqFeducials.reshape((len(RealSeqFeducials), 1), order = 'F')
x_Feducials = (RealSeqFeducials.tolist())
for i in range(len(Sequence_FeducialsList)):
    if str(Sequence_FeducialsList[i])[2:-2] == str(255):
        SequenceListFinal[i] = str(x_Feducials[0])[2:-2]
# THIS ADDS PASSIVATION PATTERN TO THE ARRAY
Layout = Image.open("SeqDepF_Layout.bmp")
Layout = np.array(Layout)
grid_Layout = (Layout.reshape((786432,1), order = "F"))
np.savetxt("single_line_Layout.txt", grid_Layout, delimiter="", fmt='%5.0f')
Sequence_Layout = np.array(grid_Layout)
Sequence_Layout = Sequence_Layout.astype("str")
Sequence_LayoutList = Sequence_Layout.tolist()
RealSeqLayout = np.loadtxt("SelectedLayout.txt", dtype=str)
RealSeqLayout = RealSeqLayout.reshape((len(RealSeqLayout), 1), order = 'F')
x_Layout = (RealSeqLayout.tolist())
for i in range(len(Sequence_LayoutList)):
    if str(Sequence_LayoutList[i])[2:-2] == str(255):
        SequenceListFinal[i] = str(x_Layout[0])[2:-2]
# THIS CREATES INPUT FOR THE MATLAB
with open('SeqForMatlab8bit.txt', 'w') as file_handle:
    for list_item in SequenceListFinal:
        file_handle.write('%s\n' % list_item)
print("Job Done!")
```

Scripts – Flash_v3 Microarray Data Organiser

```
import glob, os
import pandas as pd
import numpy as np
probe_list =[]
for file in glob.glob("*.probe"):
    probe_list.append(file)

Data = pd.read_csv(str(probe_list)[2:-2], delimiter= '\t', skiprows=1, usecols=[1,8])

Fixed_Data1 = Data.groupby(Data.columns[0]).mean()
Fixed_Data1 = Data.groupby(Data.columns[0])[Data.columns[1]].agg(Average="mean", StandardErrorOfAverage= "sem",
StandardDeviationOfSample= "std", Median= "median")
Fixed_Data1.to_csv("Flash_" + str(probe_list)[2:-8] + ".csv")
print("Job Done!")
```

9. Supplementary figures

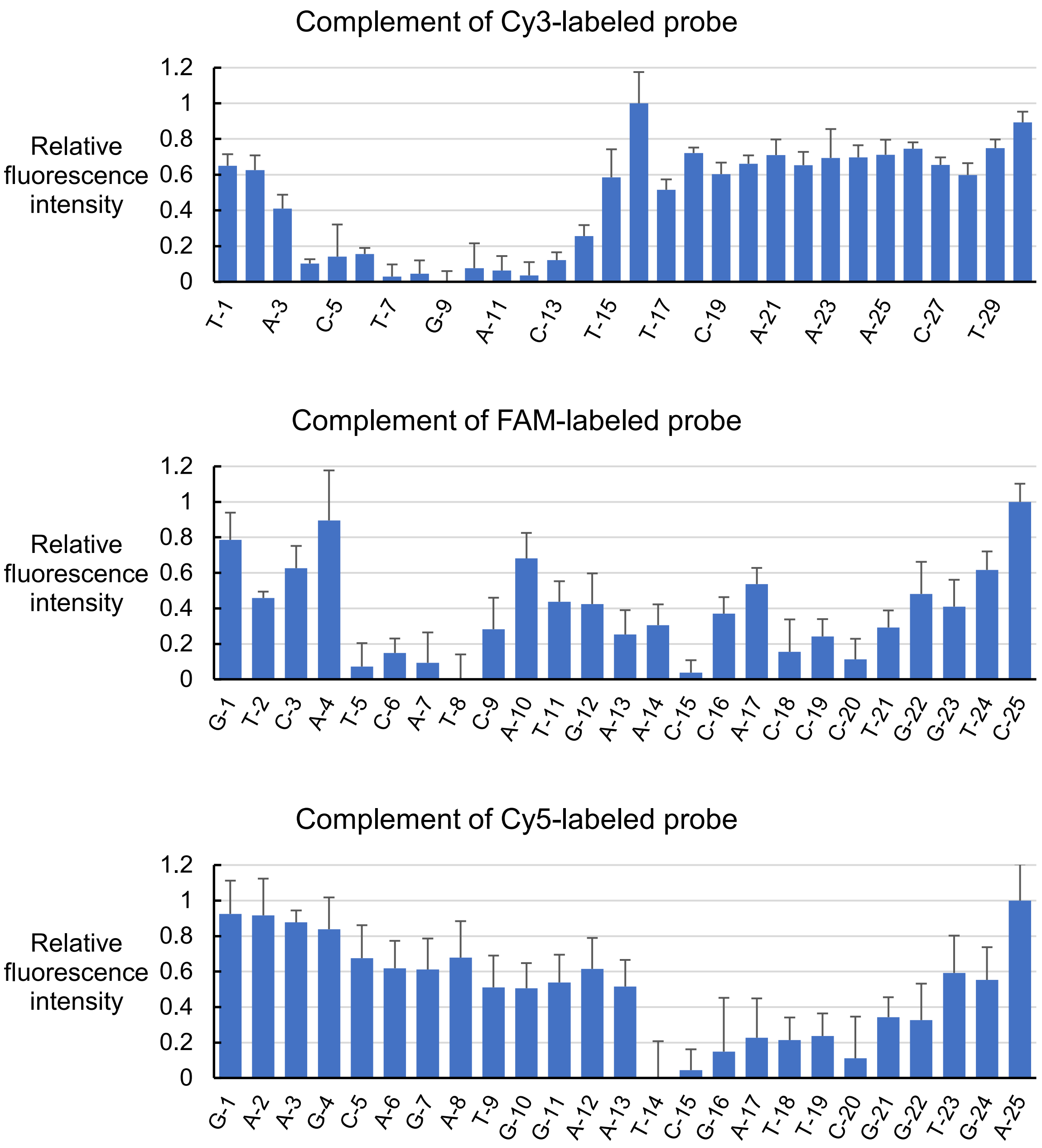

Figure S1. Average effect of truncation on hybridization signal to the Cy3, Cy5 and FAM-labeled probe. Data is presented relative to full-match hybridization signal (set to 1) and for each truncated position along the DNA sequence synthesized on the microarray (in the 5′-3′ direction). 0 indicates background fluorescence.

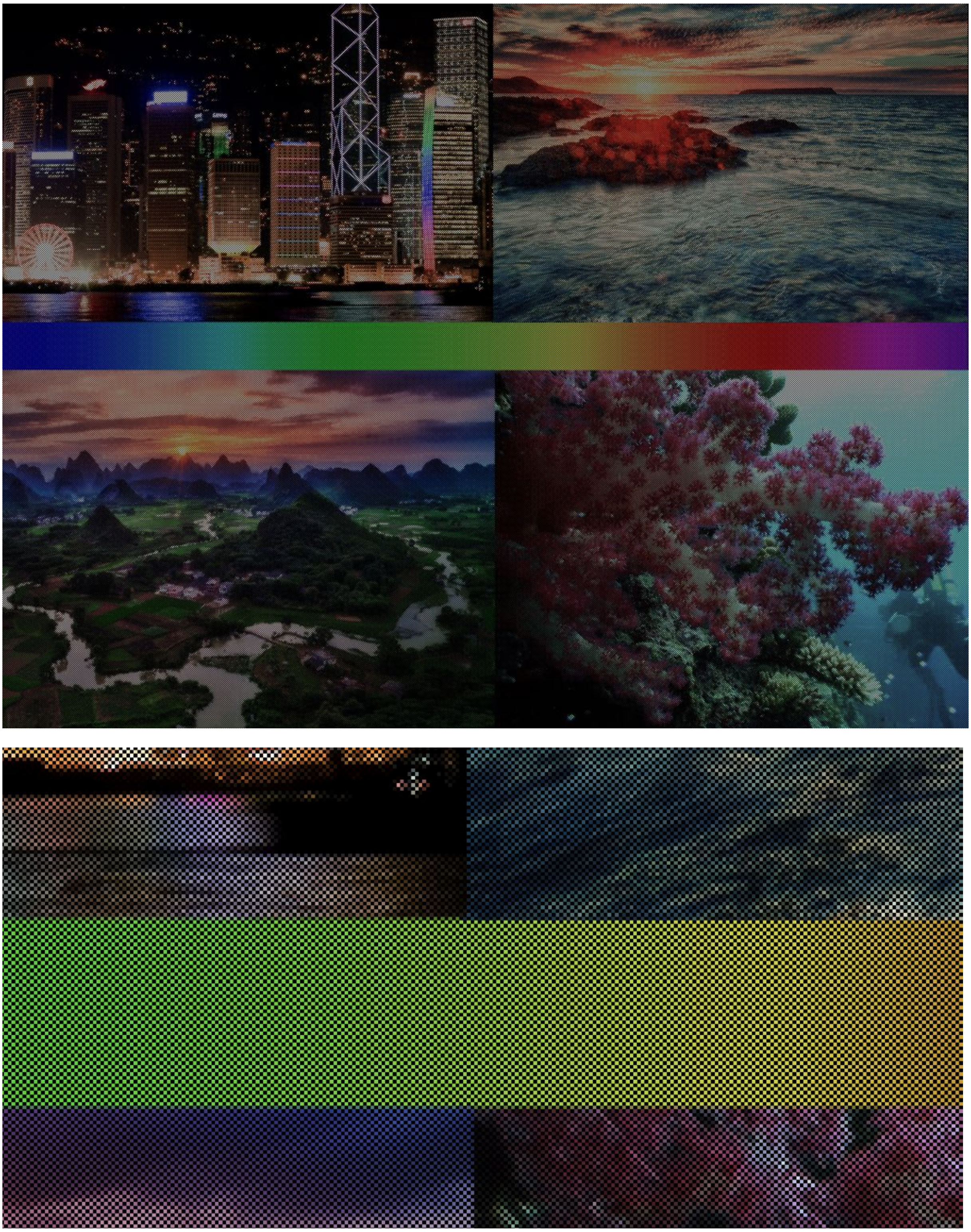

Figure S2. (top) Input 24-bit image consisting of a collage of four landscape/wildlife pictures separated by a color gradient. Original resolution is 1024 × 768 and corresponds to DMD resolution in our MAS setup. (bottom) A checkered pattern has been placed atop the collage to create a grid of black pixels. Black pixels correspond to mirrors tilted in an "OFF" position. "OFF" mirrors redirect UV light away from the surface, leaving the corresponding features non-exposed. This grid-like design separates illuminated features in a diagonal manner so that neighbouring features only have corner-to-corner contact and it significantly reduces non-intended photodeprotection during exposure to UV. In turn, this precaution increases DNA synthesis quality throughout the microarray.

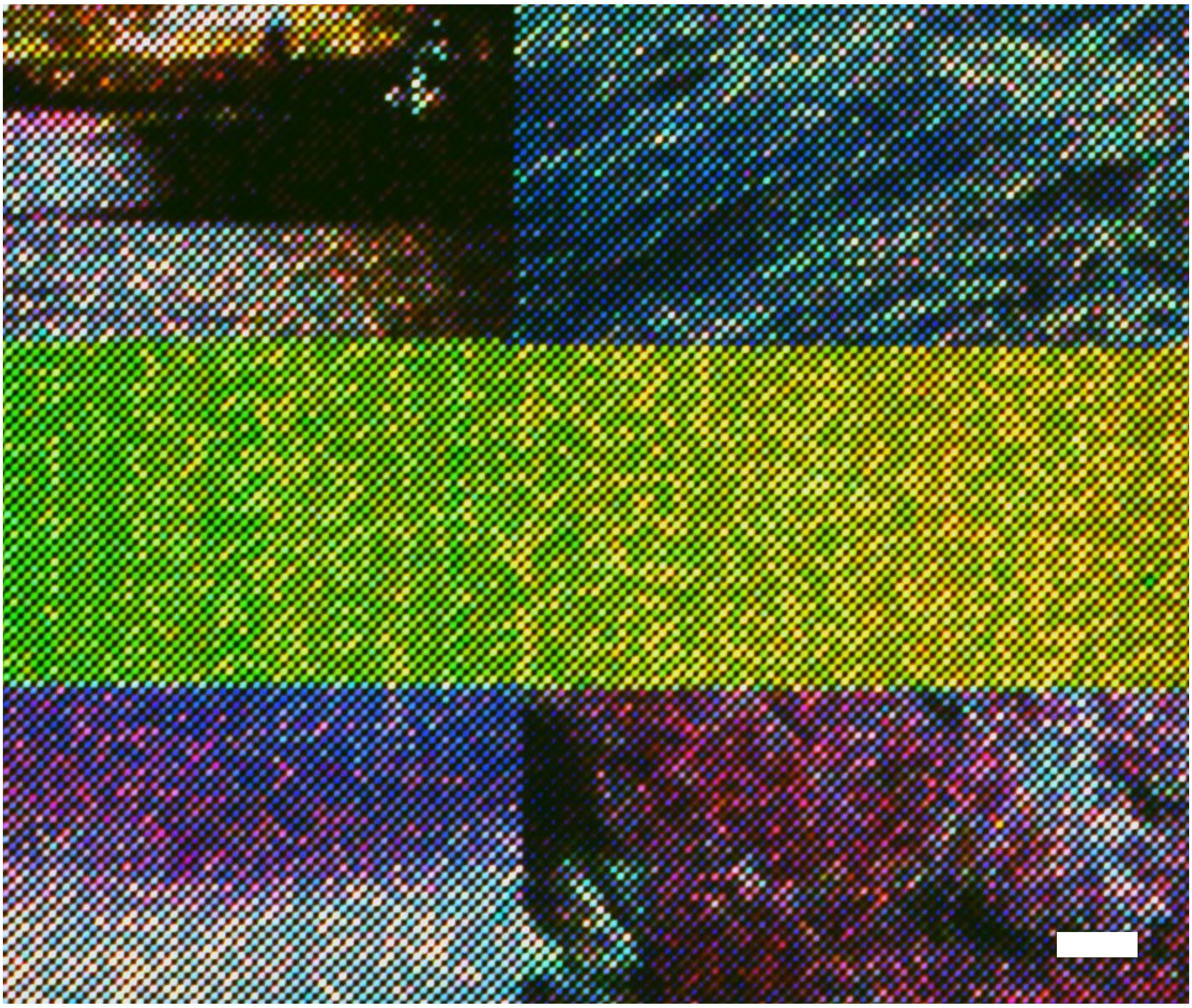

Figure S3. Cropped-out, zoomed-in version of the output image with merged RGB channels. Original resolution is 5658 × 4283 (2.5 µm resolution scan of a 1.4 × 1 cm synthesis area). Scale bar is ~200 µm.

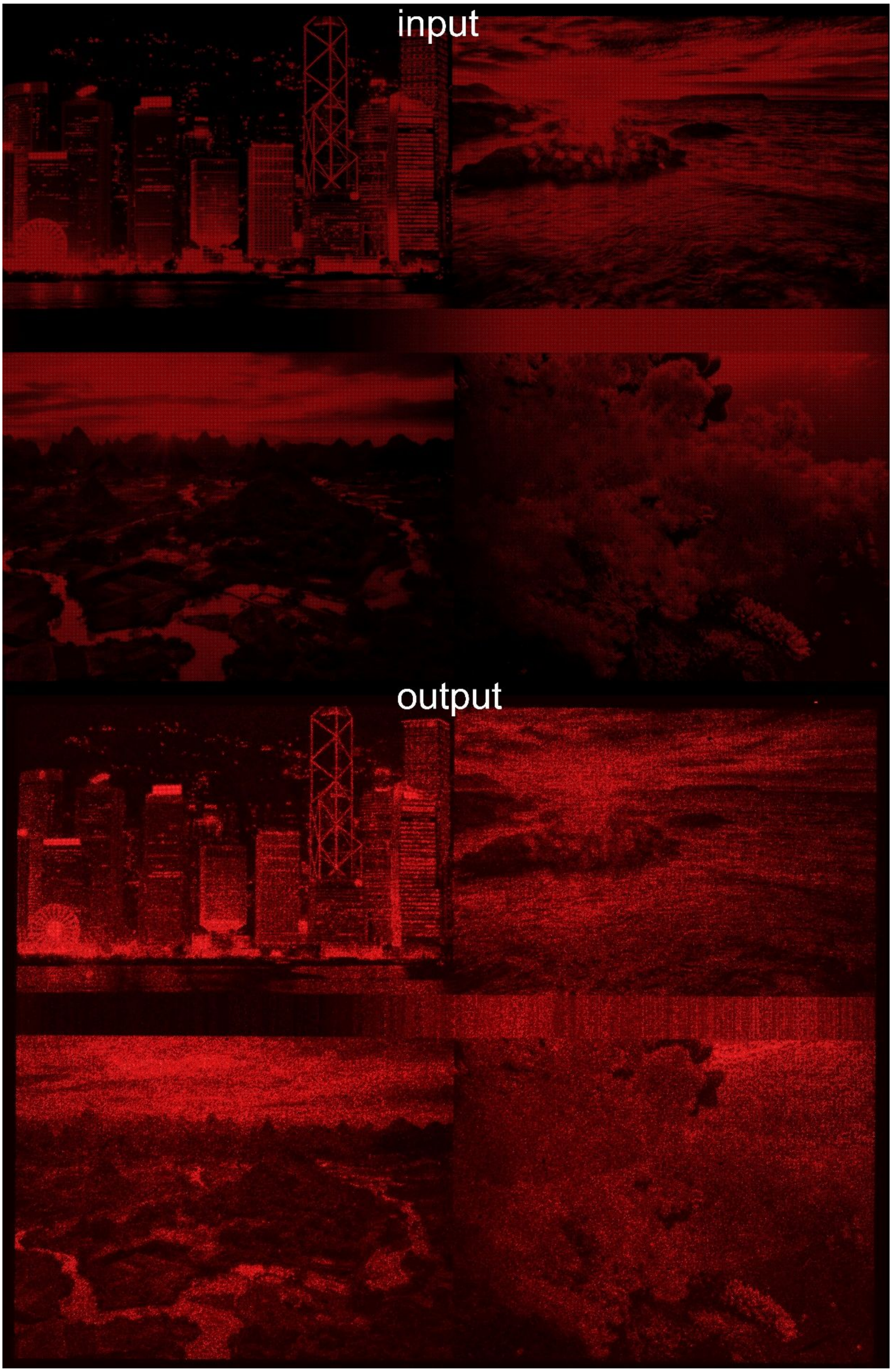

Figure S4. Decomposed RGB channels in input and output images. Red channel in 256-color grayscale (input, top) and Cy5 scan after hybridization to the Cy5-labeled probe. The microarray was scanned at 2.5 μm resolution using 635 nm laser excitation.

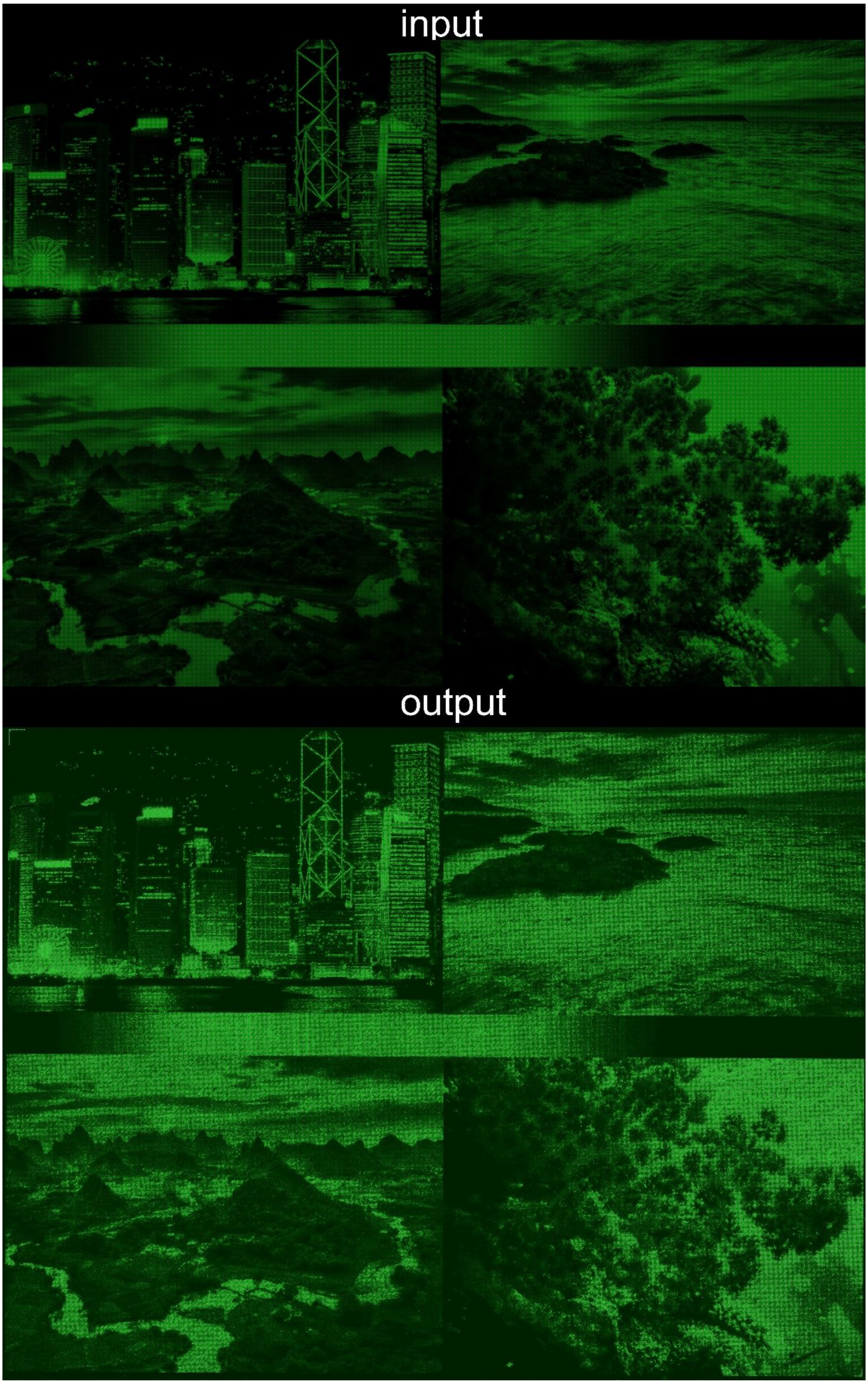

Figure S5. Decomposed RGB channels in input and output images. Green channel in 256-color grayscale (input, top) and Cy3 scan after hybridization to the Cy3-labeled probe. The microarray was scanned at 2.5 µm resolution using 532 nm laser excitation.

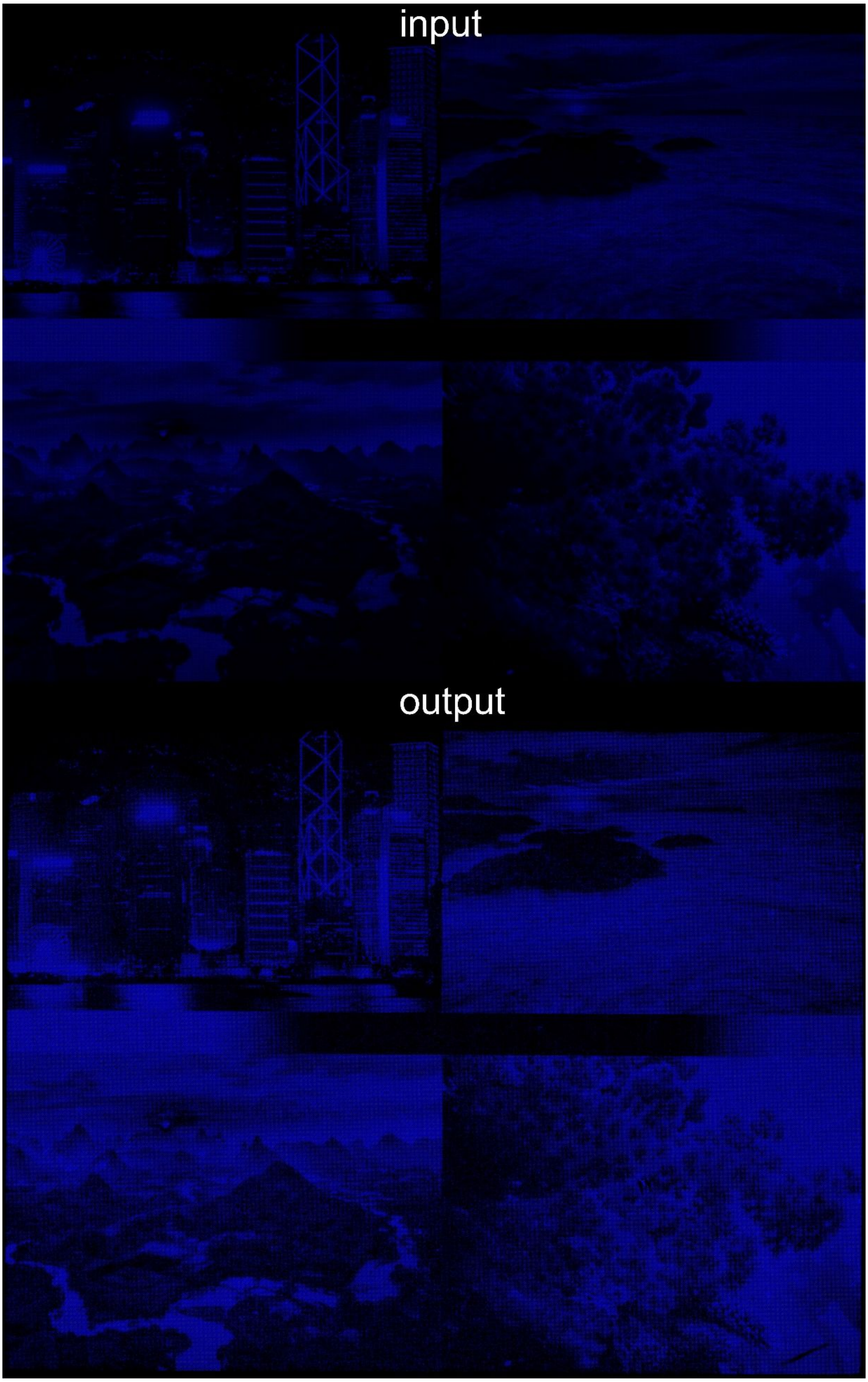

Figure S6. Decomposed RGB channels in input and output images. Blue channel in 256-color grayscale (input, top) and fluorescein scan after hybridization to the fluorescein-labeled probe. The microarray was scanned at 2.5 µm resolution using 488 nm laser excitation.
