# Supplementary figures and images for: A Canvas of Spatially Arranged DNA Strands that Can Produce 24-bit Color Depth
Source: J Am Chem Soc. 2023 Oct 3;145(41):22293–7. doi: 10.1021/jacs.3c06500 (PMC10591465; doi:10.1021/jacs.3c06500)

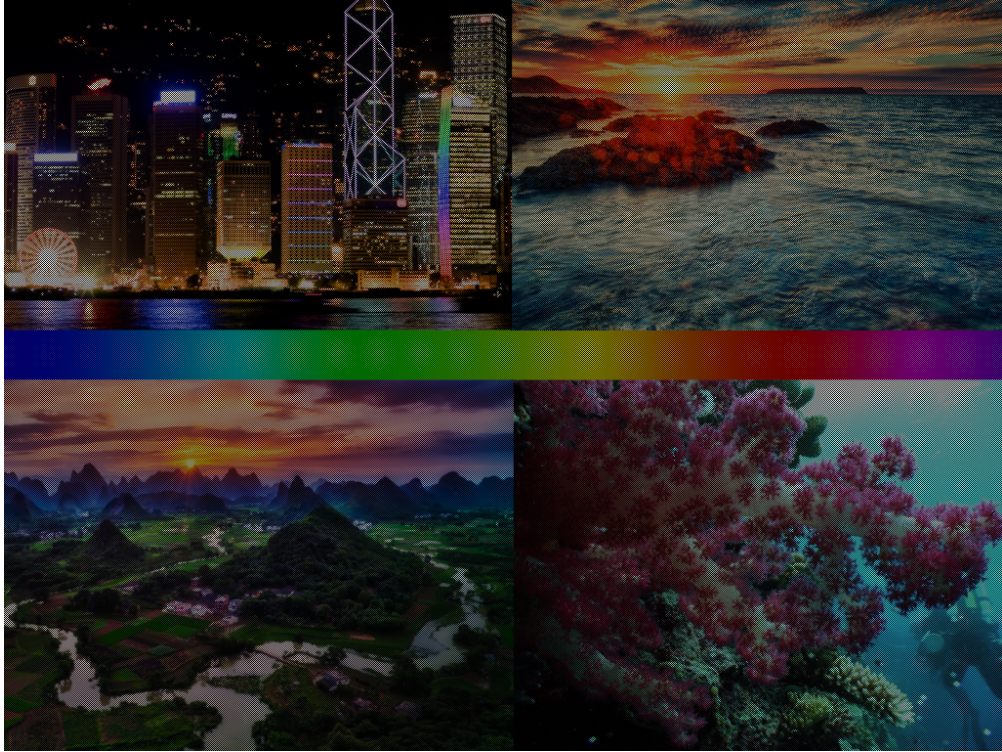

86x65mm (300 x 300 DPI)

Supplement: Supplementary file 3 — ja3c06500_si_003.pdf [file ja3c06500_si_003.pdf]
